# Developing a Research Instrument to Document Awareness, Knowledge, and Attitudes Regarding Breast Cancer and Early Detection Techniques for Pakistani Women: The Breast Cancer Inventory (BCI)

**DOI:** 10.3390/diseases4040037

**Published:** 2016-12-09

**Authors:** Atta Abbas Naqvi, Fatima Zehra, Rizwan Ahmad, Niyaz Ahmad

**Affiliations:** 1 Department of Pharmacy Practice, College of Clinical Pharmacy, University of Dammam, Dammam 31441, Saudi Arabia; 2 Department of Social Sciences, Shaheed Zulfikar Ali Bhutto Institute of Science and Technology SZABIST, Clifton, Karachi 75600, Pakistan; zehra.fatima90@gmail.com; 3 Natural Products and Alternative Medicines, College of Clinical Pharmacy, University of Dammam, Dammam 31441, Saudi Arabia; rareriyadh@ud.edu.sa; 4 Department of Pharmaceutics, College of Clinical Pharmacy, University of Dammam, Dammam 31441, Saudi Arabia; nanhussain@ud.edu.sa

**Keywords:** breast cancer, research methodology, reliability, validation, piloting, pretesting, questionnaire

## Abstract

There is a general hesitation in participation among Pakistani women when it comes to giving their responses in surveys related to breast cancer which may be due to the associated stigma and conservatism in society. We felt that no research instrument was able to extract information from the respondents to the extent it was needed for the successful execution of our study. The need to develop a research instrument tailored for Pakistani women was based upon the fact that most Pakistani women come from a conservative background and sometimes view this topic as provocative and believe discussing publicly about it as inappropriate. Existing research instruments exhibited a number of weaknesses during literature review. Therefore, using them may not be able to extract information concretely. A research instrument was, thus, developed exclusively. It was coined as, “breast cancer inventory (BCI)” by a panel of experts for executing a study aimed at documenting awareness, knowledge, and attitudes of Pakistani women regarding breast cancer and early detection techniques. The study is still in the data collection phase. The statistical analysis involved the Kaiser-Meyer-Olkin (KMO) measure and Bartlett’s test for sampling adequacy. In addition, reliability analysis and exploratory factor analysis (EFA) were, also employed. This concept paper focuses on the development, piloting and validation of the BCI. It is the first research instrument which has high acceptability among Pakistani women and is able to extract adequate information from the respondents without causing embarrassment or unease.

## 1. Introduction

Breast cancer can be defined as an uncontrolled growth in the cells of the breast tissue. The cells continue to grow abnormally resulting in a malignant tumor in the breast. The tumor then grows over time. Initially the tumor remains localized but can also spread to other parts of the body with time [1,2].

It is becoming a major health concern in Pakistan. The country has the highest incidence of breast cancer in the region. Studies report that one (1) in every nine (9) women in Pakistan will develop breast cancer in her life [3]. Moreover, Pakistan lacks a dedicated health care agency to collect exact figures of cancers, particularly breast cancer cases, in Pakistan [4]. The Karachi Cancer Registry^®^ has been the only recognized cancer data registry until now. It collected data from the south Karachi district which is about 1% of the total population of Pakistan. Cancer data from the Karachi Cancer Registry^®^ reported a third (34.6%) of total cancers occurring in women to be breast cancer during 1995–2003 [5]. Recently, the latest report from the largest cancer hospital in the country covering data from December 1995 to December 2009 was published. It highlighted almost half (45.9%) of the total cancers among females to be of breast origin [4].

Few studies have been carried out in this regard in Pakistan. It has been reported that Pakistani women present breast cancer in its later stages when it becomes very difficult to treat the ailment or, sometimes, the cancer is incurable. The reasons for this delayed presentation are numerous, such as financial issues and low attribution of symptoms. Apart from low awareness and knowledge of women, conservatism and high stigma towards the subject hinders breast cancer awareness and screening [6,7,8,9].

A study conducted among female patients attending a hospital in the city of Rawalpindi to assess knowledge, attitudes, and practices towards breast cancer had a large sample size and used stratified random sampling. Additionally, it described the sampling method in detail. It employed a pre-tested, well-defined research instrument distributed in three different sections related to knowledge, attitudes, and practices, which was easy to understand. However, the pre-testing and validation of the questionnaire was undefined. The study did not grade knowledge in terms of scores [7]. Similarly, another study conducted in the city of Lahore tested knowledge and attitudes of females towards breast cancer. The respondents were attending a hospital either as patients or caregivers. The study reported inadequate knowledge of the respondents regarding the ailment. Despite a large sample size, the study had some limitations. It was conducted in a hospital and the target audience was not the general public. The research instrument had questions which were difficult to comprehend for the general public. The questionnaire had scoring criteria for knowledge; however, the study did not explain the standards against which the responses were graded as correct or wrong. The study was designed as cross-sectional and its duration was about one year [8].

Another study conducted among women admitted in a tertiary care hospital in Lahore revealed lack of awareness and knowledge regarding breast cancer. The study was conducted on a small scale with a small sample size. The questionnaire was interview-based [9]. Similarly, a study conducted in a gynecology unit of hospitals in a city in Pakistan tested knowledge regarding risk factors of breast cancer. The respondents were the female patients seeking medical care in OPD related to gynae and obstetrics. The sample size was very small i.e., (N = 100) and the study had a limited scope as it only included knowledge about risk factors of breast cancer. Scoring criteria was defined, however, no standards were explained [10]. Another study conducted in the city of Karachi also had a small sample of 373 patients who were attending clinics. The questionnaire was not intelligible for the whole population. Those who could not comprehend were simply excluded from the study. The pre-testing procedure of the research instrument was not described. Though, the authors mentioned that some issues were addressed during pre-testing, however, the details were not available regarding the nature of the problems and how they were addressed. The authors admit the research instrument had a limitation of a suggestive nature of the questions. Scoring criterion was defined, however, no standards were explained [11].

To summarize the above findings, only a few research studies have been conducted in Pakistan on the subject. All studies targeted female respondents who were available in a medical facility seeking treatment. The target population encompassing women visiting the health care facility was not representative as studies reported that not all Pakistani women sought treatment or medical advice regularly. As a result of such sampling, a large target population was left out [12]. Moreover, this positive health seeking attitude can lead to a greater chance of knowledge-gathering. Hence, the response from such a target segment cannot be generalized to the whole Pakistani female population. None of the studies described their standards and the methods used to designate the responses as correct or wrong. Most importantly, the pre-testing and validation details and procedures were not mentioned.

Taking into account the limitations in the previous literature, a more comprehensive study was designed to document awareness, knowledge, and attitudes towards breast cancer and early detection techniques in Pakistani women. There is a general hesitation in participation among the Pakistani women when it came to giving their response to the surveys on such subjects. Hence, the investigators felt that no existing research instrument was able to extract information from the respondents to the extent it was needed for the successful execution of such a study. For this purpose, a research instrument coined as the “Breast Cancer Inventory (BCI)” was especially developed for the task. It was aimed at obtaining more acceptance among Pakistani women and higher response rates and, at the same time, successfully documenting the responses regarding their awareness, knowledge, and attitudes towards breast cancer and early detection techniques. The main study is currently in progress. This concept paper focuses on the development and validation of BCI in detail.

## 2. Methods

A research instrument for the purpose of documenting information such as awareness, knowledge, and attitudes related to breast cancer and early detection techniques was developed by a panel of experts specifically for Pakistani women. The research instrument was validated by piloting and statistical evaluation.

### 2.1. Rationale

The need to develop a research instrument tailored for Pakistani women was based upon the fact that most Pakistani women come from a conservative background and sometimes view this topic as provocative and believe discussing publicly about it as inappropriate. Additionally, a review of the available literature revealed that existing research instruments had many limitations. Therefore, using such research instruments might not be able to extract their information concretely.

### 2.2. Expert Panel

A panel was set up to discuss breast cancer and prevention techniques and design an appropriate study for documenting the required information specifically for Pakistani women. The panel consisted of three practicing medical professionals (one male and two females) specializing in oncology and gynecology, respectively, with experience over 10 years, a clinical pharmacist specialized in patient counseling and pharmaceutical care practicing for more than six years in an oncology ward, and an occupational therapist dealing with such patients for five years. The other non-medical experts included a university professor specialized in clinical pharmacy and research methodology with a work experience of over 15 years, two social workers with degrees in social sciences and field experience of three years were also a part of the panel.

### 2.3. Literature Review and Discrepancies in the Existing Research Instruments

After the formulation of an expert panel, a literature search was carried out. Only a few research studies have been conducted so far on the subject. The results of the literature review revealed that five existing dedicated research instruments for Pakistani women were available. The panel analyzed each instrument for its suitability and reproducibility. Unfortunately, all studies target a particular segment of the population i.e., Pakistani women presenting in health care facilities seeking treatment. Hence, the questions were tailored for such respondents who have some level of understanding and awareness as compared to those who do not seek medical advice/treatment that often.

The panel deemed some research questions difficult to understand and the use of difficult medical terminologies such as ‘nulliparity’ and jargons such as ‘BSE’. The demographic profile was limited to a few variables. Important socioeconomic variables, such as family income, occupation, number of children, etc., were absent. Some research instruments had a limited scope to test knowledge and/or awareness, such as focusing only on the knowledge and/or awareness about risk factors of breast cancer. None of the research instruments identified standards and had their piloting and validation procedures mentioned.

The research instrument had binary outcomes, i.e., (Yes/No). They fail to assess the degree of knowledge/awareness regarding the issue i.e., a respondent having a certain degree of awareness/knowledge and a respondent having no awareness/knowledge could not be differentiated. Moreover, some of the research instruments had a limitation of a suggestive nature of questions and elements of chance [7,8,9,10,11]. None of the existing dedicated research instruments were deemed fit for use by the panel.

### 2.4. Conceptualization of Research Instrument

The conception of BCI started by formulating a panel of experts and, subsequently, conducting a detailed literature search to collect background information on the subject. The literature search reviewed all available studies and existing research instruments on the subject. Moreover, in depth interviews were conducted by the panel with 32 women. This was done to identify and assess the items which had the potential to become research questions. The panel assessed awareness, knowledge, and attitudes of Pakistani women as potential areas to investigate. Thus, the questionnaire was conceptualized to document information about awareness, knowledge, and attitudes of Pakistani women. The BCI was formulated in English and Urdu versions using language which was easy to understand. Moreover, the knowledge section of the research was designed as a scoring scale to quantify the knowledge of respondents on a scale of 0–22 points. Standards were also identified to compare/designate the responses as correct or wrong. The questionnaire was initially designed to be interview-based however; it was modified to be self-reporting after pilot results.

### 2.5. Standards

The standards identified were National Breast Cancer Foundation, USA and Canadian Cancer Society [13,14].

### 2.6. Design of the BCI

The BCI was divided into four different sections. Section 1 of the questionnaire consisted of demographic variables, whereas Sections 2–4 dealt with awareness, knowledge, and attitudes, respectively. The demographic variables included age, marital status, number of children, education, occupation, family income, and ethnicity. Furthermore, questions related to any person or family member with breast cancer was also added.

Section 2 of the BCI focused on awareness related to breast cancer and detection techniques. Questions related to awareness regarding breast cancer, screening for breast cancer, physical self-examination for detecting BC, mammography for BC, and treatment were added. Later, questions for documenting the source of information regarding BC were also added. All questions related to awareness had three possible options i.e., “Yes”, “No”, and “Yes, but I am not sure”.

Section 3 dealt with the attitudes. Questions related to personal opinions regarding screening, possible reason to undergo screening if need be, feelings regarding discussing BC in public and with a physician, and gender preference in selecting a health care provider in this context were added. Further to this, questions related to perceived barriers to screening for BC, advice to other women regarding screening, and personal viewpoints regarding BC as a health care threat for Pakistani women were also included.

Section 4 of BCI was aimed at assessing the knowledge regarding BC and early detection methods. For this purpose, research questions investigating if Pakistani women knew about BC, its most common symptoms, and risk factors were included. Moreover, questions related to metastasis of breast cancer and overweight/obesity increasing the chances of having BC were added. Section 4 also included questions regarding availability of BC screening and provision for physical self-exam. Lastly, those who answered positively to the last question were further asked if they know how to conduct physical self-exam. All questions except those inquiring about risk factors and symptoms had three possible options i.e., “Yes”, “No”, and “Yes, but I am not sure”. These two questions were formulated as open-ended to eliminate the element of chance and suggestive nature as observed in existing instruments.

### 2.7. Item Scoring in the Knowledge Domain

In the knowledge section of the BCI, the responses for each research question carried a score. There were 10 research questions in the BCI knowledge domain and each item had a score. The standards identified for comparing answers mentioned by the respondents were from the Canadian Cancer Society and the National Cancer Institute, USA. The grading criteria were defined by the panel of experts. For every response designated as ‘correct’ had a score of two (2) and an answer designated as ‘wrong’ was marked zero (0). For the questions related to risk factors and symptoms of breast cancer, the grading was a bit complex. Three (3) points were awarded if the answer was an established symptom/risk factors according to identified standards AND/OR for any two (2) or more answers all of which are established symptoms/ risk factors for breast cancer. Two (2) points were given for any (2) answers one (1) of which is established symptom/risk factor according to identified standards and the other is not established but had a likelihood. Further to this, one and a half (1.5) points were awarded if (2) answers were given one of which is established symptom/risk factors and the other is a wrong answer OR (1) or more answers both of which are not established symptom/risk factors but had a likelihood i.e., not a wrong answer. Lastly, Answers which are neither established according to identified standards nor possess a likelihood i.e., wrong answers, were given zero (0) points.

The details of the grading are tabulated in Supplementary Materials Table S1.

### 2.8. Scoring Range in the Knowledge Domain and Categories

The scoring range of the knowledge domain of the BCI was from 0–22 points. The individual score of each research variable was summed to yield a final score. The final score obtained was graded into four possible categories, i.e., very low knowledge, low knowledge, adequate knowledge, and excellent knowledge. The summary of grading criteria is tabulated in Table 1.

### 2.9. Respondents’ Time of Choice

The respondents’ time of choice as observed from the pilot study were morning hours and early evening hours.

### 2.10. Participants

All Pakistani women older than 18 years, without active or past history of breast cancer were included in the study. All Pakistani women below 18 years, women suffering from breast cancer or had past history, and women having dual nationality were excluded.

### 2.11. Sampling Procedure

The sampling procedure employed was stratified convenience sampling. Pakistani women were first divided into three strata i.e., employed, unemployed, or housewife and students, after which convenience sampling was employed. Employed women were approached in firms and also contacted through emails displayed in the firm’s employee directory displayed on their website. Professional networking sites were also used to contact employed women. Students were approached in the universities during ‘off peak’ hours and unemployed females were approached by social workers at their place. After their consent, the questionnaire was handed to the respondents and received at their time of choosing.

### 2.12. Duration of Pilot Study

The duration of pilot study was from March 2015 to May 2015.

### 2.13. Pilot Study and Validation Process

The BCI was initially piloted in 25 women. It took an average 17 minutes to fill in the responses. The pilot results of the first draft of BCI termed as BCI-I, which consisted of 33 research questions, revealed a low acceptability. The response rate achieved was 48% (N = 12/25). Moreover, those participants who consented to give their response were hesitant to answer some of the questions. In addition, many respondents were not comfortable in giving their responses in an interview-based setup to a male investigator. The participants who were interviewed in the morning hours seemed more enthusiastic.

The results of the pilot study were presented to the panel of experts and discussed. After the first pilot study, and subsequent discussion with the panel, changes were made to the draft of BCI. The interview-based setup was undertaken by female investigators. A research question, i.e., “Did you notice any change in your breasts,” was deleted from the BCI. The time for the survey was identified as morning hours and early evening hours.

The second draft of the BCI, termed as BCI-II, was piloted in 12 women. This time though, a response rate of 58.3% (N = 7/12) was achieved; however, some of the respondents seemed uncomfortable to participate in the interview-based setup despite having a female investigator. The reason given by the respondents for refusal was a busy schedule. Apart from this, some participants belonged to a specific ethnicity and a family income range which was not mentioned in the instrument. In research questions relating to the number of children, there was a single category for participants who were single and married with no children. Hence, there was no discrimination between them. Unmarried parenting is not practiced in Pakistan; hence, participants who were married with no children and respondents who were single could not be differentiated. There was a tendency to skip the question of religious views as some of the questionnaires returned had the item unmarked. Only those who observed the Islamic school of thought marked their choice. The second draft was again subjected to the panel for discussion and further modification.

The BCI-II was again modified. The interview-based questionnaire was changed to a self-reported form. Categories in ethnicity and family income were increased. In the item for the number of children, a separate category i.e., “Not applicable (if single)” was added in the questionnaire. The panel felt the research item of “Religious views” was somewhat biased in nature and recommended it to be removed from BCI-II to III.

The third draft of the BCI, i.e., BCI-III, was piloted again in 23 women. It achieved a response rate of 82.6% (N = 19/23). This time however, a higher acceptability was achieved. After the pilot results, it was observed that the research questions relating to most common symptoms and risk factors of breast cancer had a suggestive nature. Since the questionnaire was changed to a self-reported form, women who had a low level of education could not comprehend the English language. The BCI-III was again modified since the panel suspected that the closed-ended form of research question for symptoms and risk factors could not assess the knowledge regarding the subject. The two questions were modified to open-ended and all possible options were removed. The respondents’ answer was checked by standards. An empty box, i.e., no answer, was designated as “Do not know”. It was graded as zero (0). An Urdu version of BCI was also prepared.

The fourth draft was coined as BCI-4 and was piloted again in nine women. The response rate was 100% (N = 9/9) and no discrepancy was observed this time. However, it was observed that some of the respondents mentioned symptoms and risk factors of breast cancer which do possess likelihood but are not established. Furthermore, some respondents mentioned more than one symptom and risk factor. The problem was resolved by setting up grading criteria as highlighted in Table S1.

**Figure 1:**
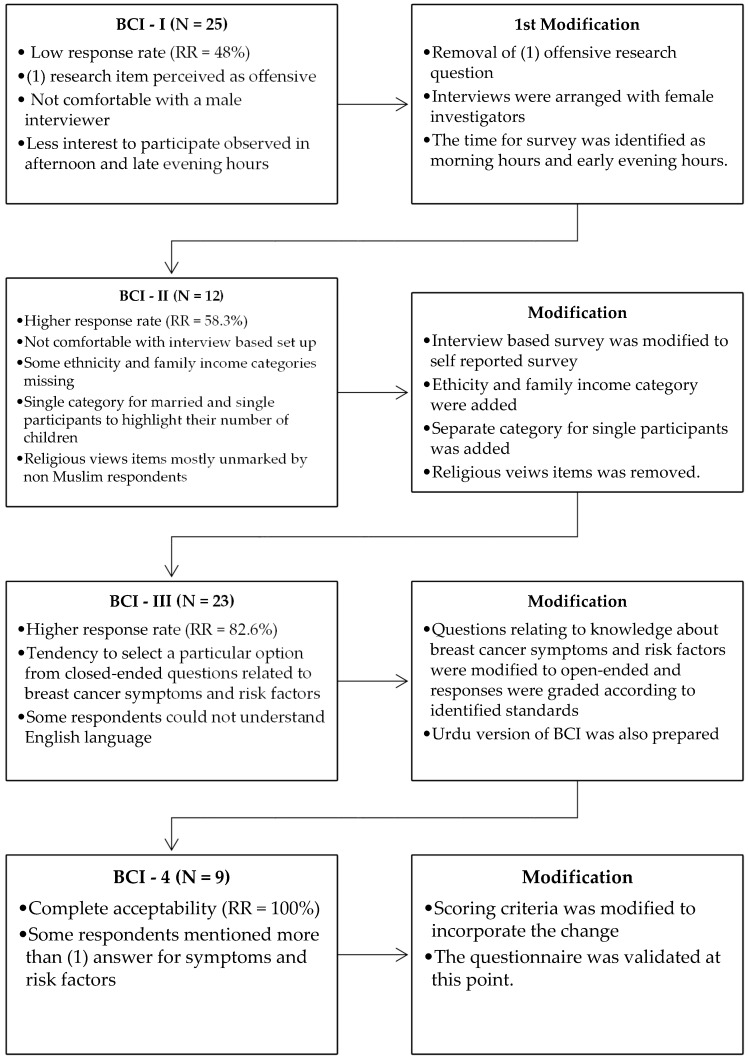
Summary of the BCI construction and validation process.

The BCI-4 was validated and was finally approved by the panel of experts. The duration of piloting of each version of BCI was one day i.e., the investigators handed the BCI on a single day to the target population who consented to participate. Hence, the sample for each pilot result is different. The responses were received in a span of 1–8 working days. No respondent was repeated throughout the piloting of all four (4) versions of the BCI. A flow chart of questionnaire construction and validation process is presented in Figure 1.

### 2.14. Ethical Approval and Participants’ Consent

This concept paper is a part of a research study aimed at documenting the awareness, knowledge, and attitudes of Pakistani women regarding breast cancer and early detection. The study is ethically approval by the Department of Oncology, Central Hospital, Karachi 75600, Pakistan (letter #2015-2) and the Institutional Review Committee (IRC) of Clifton Hospital, Karachi 75600, Pakistan (IRC approval #24-2015).

The participants were informed about the study and their consent was obtained before being handed the questionnaire. Participation was voluntary and neither the participants were forced to participate nor were they offered any incentive. Moreover, the participants’ identity and personal information were not asked and/or kept confidential.

## 3. Results

### 3.1. Demographics of the Participants Selected for Initial Interviews

The initial pool of participants who consented for in-depth interviews were mostly in the age group between 18 and 30 years (N = 20, 62.5%) followed by slightly less than a quarter (N = 7, 21.8%) in the age group between 31 and 41 years. Almost half of the participants appeared single (N = 16, 50%). The majority of the participants (N = 16, 50%) had education equal to higher secondary or more, and slightly less than half of the target segment (N = 13, 40.6%) was students. The details of demographics are tabulated in Table 2.

### 3.2. Response Rate of BCI

**Figure 2:**
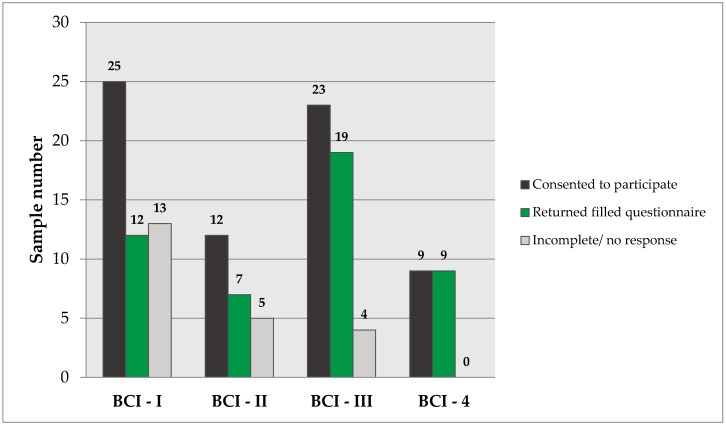
Response rate of BCI.

A total of 12 respondents out of 25 participants who consented for the piloting of the first draft of the BCI returned their filled questionnaire giving a response rate of 48%. The second draft of BCI was piloted in 12 participants after consent and achieved a response rate of 58%. The third draft of BCI achieved a response rate of 82.6%, whereas the fourth draft achieved a 100% response rate. Each progressive draft of BCI exhibited an increased degree of uptake and successful completion/return. The acceptability of all versions of BCI, in terms of sample number, is graphically represented in Figure 2.

### 3.3. Demographics of the Participants of BCI-I Pilot Study

The respondents involved in the piloting of the first draft of the BCI were mostly (N = 5, 50%) between 18 and 30 years old with respect to their age and appeared married (N = 7, 58.3%) for the most part. Almost half of the participants (N = 6, 50%) were educated up to higher secondary school (intermediate level). Regarding their occupation, a quarter of the target segment (N = 3, 25%) seemed involved in household activities and a third proportion (N = 4, 33.3%) was studying. Less the half (N = 5, 41.7%) were employed. A third proportion (N = 4, 33.3%) were of Urdu-speaking ethnicity, followed by a quarter (N = 3, 25%) of participants belonging to Punjabi origin.

### 3.4. Demographics of the Participants of BCI-II Pilot Study

In the pilot study of the second draft of BCI, the respondents were mostly in age groups between 18 and 30 years (N = 3, 42.8%) followed by equal proportions of participants in age groups between 31 and 40 years and above 40 years i.e., (N = 2, 28.6%). The majority appeared (N = 4, 57.1%) to be in a marital relationship with equal number of participants having completed their education up to higher secondary school (intermediate level) and graduation i.e., (N = 3, 42.9%). Similarly, an equal proportion of respondents (N = 3, 42.9%) appeared to be employed and studying. A majority (N = 4, 57.1%) were of Urdu-speaking origin.

### 3.5. Demographics of the Participants of BCI-III Pilot Study

The majority of the participants taking part in the pilot study of the third draft were in the age group between 18 and 30 years i.e., (N = 8, 42.1%), followed by some respondents (N = 7, 36.8%) between 31 and 40 years. The majority of the respondents were single and had an education up to higher secondary school (intermediate level) i.e., (N = 9, 47.4%). Most of them were students (N = 7, 36.8%) followed by equal proportions of participants being employed and involved in household activities i.e., (N = 6, 31.6%). The majority were of Urdu-speaking ethnicity (N = 7, 36.8%) followed by Punjabis (N = 4, 21.1%).

### 3.6. Demographics of the Participants of BCI-4 Pilot Study

Almost half of the respondents (N = 4, 44.5%) were in age between 18 and 30 years, and more than half (N = 6, 66.7%), were married. An equal number of participants appeared having completed their education up to the level of secondary school (matriculation) and higher secondary school (intermediate level) i.e., (N = 3. 33.3%). In terms of occupation, most of the respondents (N = 4, 44.4%) were students. The majority were Urdu speakers (N = 3, 33.3%) followed by equal proportion of Sindhi, Pushtun, Baloch, Punjabis, and other ethnicities, i.e., (N = 1, 11.1%). A detailed account of demographics of participants involved in the pilot studies of all four versions of BCI is tabulated in Table 3.

## 4. Sampling Adequacy, Significance, and Reliability

The sampling was adequate and the Kaiser-Meyer-Olkin measure (KMO) was reported at 0.687 and Bartlett’s test reported a significant *p*-value < 0.01. The reliability analysis reported an overall Cronbach’s alpha value of 0.705.

## 5. Exploratory Factor Analysis (EFA)

The BCI research instrument was subjected to exploratory factor analysis using IBM SPSS, version 19 (Statistical Package for Social Sciences, IBM Corporation, Armonk, New York 10504-1722, NY, USA). The extraction method used for EFA was principle axis factoring and rotation method selected was Promax with Kaiser normalization. The EFA reported a seven factor structure with 30 research questions above factor loading above the threshold value of 0.2. The remaining two questions were still retained in the research instrument due to its high recommendation by supporting literatures and ability to test the knowledge of the respondents. The EFA is tabulated in Table 4.

## 6. Discussion

Breast cancer is a growing concern in Pakistan and it thrives on the ignorance of the society to discuss and highlight it. Factors that add to the debacle are not only related to low awareness and attribution, but also being a taboo in the society. It is sometimes quite difficult to talk about the issue due to high social stigma relating to the word “breasts”. Studies report a heightened perception of embarrassment and social stigma relating to a discussion about the disease [6,7,8,9]. It is evident that the overall atmosphere of Pakistan is not welcoming to the subject.

After the conception of the study, a literature survey was conducted to find an appropriate research instrument which can fit into the conservative and socially-stigmatized Pakistani model. The few studies that were conducted in the past had some limitations with respect to their scope and methodologies. Additionally, a number of discrepancies were observed in the existing research instruments during the literature review and no research instrument was found suitable. Hence, the development of a new research instrument was recommended by the panel. The new research instrument was designated as the Breast Cancer Inventory (BCI). It was able to extract adequate information from the respondents in all four domains, i.e., demographics, awareness, knowledge, and attitudes, without prompting respondents to feel any kind of embarrassment or unease. It was the first research instrument which had high acceptability among Pakistani women i.e., BCI-4 (RR 100%, N = 9). It was available as a self-reported survey form in both simplified English and Urdu languages.

It is quite pertinent to mention here that previous studies conducted on the subject used questionnaires which had research items designed as suggestive and forced choice questions and were focused for a certain group, i.e., not for general population [7,8]. However, the BCI was designed for the general population. One of the strengths of the BCI was rigorous piloting that was done to validate the instrument. A total of four (4) drafts of the BCI were piloted. Each draft was modified based on previous pilot results. The fourth draft (BCI-4) was validated and deemed suitable. During piloting we came across a number of issues, such as offensive and ambiguous research questions, missing categories for some questions, the suggestive nature of items in the knowledge domain (particularly the ones which related to the most common symptoms and risk factors of breast cancer). These issues were then resolved by modifying the questionnaire in light of the pilot results and validating the modifications by re-piloting a new draft until no shortcomings were reported. Unlike previous studies using a customized questionnaire for documenting breast cancer knowledge in Pakistani women, the BCI was hypothetically constructed, piloted, and validated. The results of the pilot study highlight that the BCI had success in all strata of Pakistani women with respect to their socioeconomic demographics.

In terms of statistics, the BCI demonstrated excellent internal consistency and reliability. It also represented hypothetical constructs adequately. Statistical evaluation of BCI included its sampling adequacy, significance, and reliability which were tested by the Kaiser-Meyer-Olkin measure (KMO), Bartlett’s test, and reliability analysis. In addition, exploratory factor analysis (EFA) and Chronbach reliability were also conducted, and demonstrated 30 questions above the threshold for EFA and good reliability, respectively.

## 7. Strengths and Limitation of the BCI

The strength of BCI is its high acceptability among all segments of Pakistani women without any embarrassment. Rigorous piloting and appropriate statistical testing makes it a maiden research instrument to document such data from Pakistani women. Perhaps the only limitation is the amount of time BCI consumes to get filled.

## 8. Conclusions

The BCI is the first research instrument tested for appropriateness in Pakistani women both with pilot studies and statistical analysis. Above all, it extracted respondents’ information with high acceptability in pilot studies, which is quite difficult to achieve in a culturally-conservative and highly-stigmatized Pakistani society.

## Figures and Tables

**Table 1:** Summary of grading criteria.

Serial Number	Score Range	Grading
**1.**	0–7	Very low knowledge
**2.**	8–13	Low knowledge
**3.**	14–18	Adequate knowledge
**4.**	19–22	Excellent knowledge

**Table 2:** Demographics of participants.

Serial Number	Demographics	Sample (N)	Percentage (%)	*p*-Value
**1.**	**Age**			*<0.05*
	Between 18 and 30 years	20	62.5	
	Between 31 and 40 years	7	21.8	
	Between 51 and 60 years	5	15.7	
	**Total**	**32**	**100**	
**2.**	**Marital Status**			*>0.05*
	Single	16	50	
	Married	15	46.9	
	Other	1	3.1	
	**Total**	**32**	**100**	
**3.**	**Education**			*<0.05*
	No formal education	0	0	
	Primary education	0	0	
	Secondary education	2	6.2	
	Higher secondary education	16	50	
	Graduation or higher studies	14	43.8	
	**Total**	**32**	**100**	
**4.**	**Occupation**			*<0.05*
	Employed	9	28.1	
	Student	13	40.6	
	House hold	10	31.3	
	**Total**	**32**	**100**	
**5.**	**Ethnicity**			*<0.05*
	Urdu speakers	19	59.3	
	Sindhi	1	3.1	
	Punjabi	6	18.7	
	Pushtun	2	6.2	
	Baloch	1	3.1	
	Others	3	9.3	
	**Total**	**32**	**100**	

**Table 3:** Summary of demographics of participants in pilot studies.

Pilot Results		BCI-I		BCI-II		BCI-III		BCI-4	
Serial Number	Demographics	Sample (N)	Percentage (%)	Sample (N)	Percentage (%)	Sample (N)	Percentage (%)	Sample (N)	Percentage (%)
**1.**	**Age**								
	Between 18 and 30 years	6	50	3	42.8	8	42.1	4	44.5
	Between 31 and 40 years	4	33.3	2	28.6	7	36.8	3	33.3
	Between 51 and 60 years	2	16.7	2	28.6	4	21.1	2	22.2
	**Total**	**12**	**100**	**7**	**100**	**19**	**100**	**9**	**100**
**2.**	**Marital Status**								
	Single	5	41.7	3	42.9	9	47.4	2	22.2
	Married	7	58.3	4	57.1	8	42.1	6	66.7
	Other	0	0	0	0	2	10.5	1	11.1
	**Total**	**12**	**100**	**7**	**100**	**19**	**100**	**9**	**100**
**3.**	**Education**								
	No formal education	0	0	0	0	1	5.2	1	11.1
	Primary education	0	0	0	0	0	0	0	0
	Secondary education	2	16.7	1	14.2	4	21.1	3	33.3
	Higher secondary	6	50	3	42.9	9	47.4	3	33.3
	Graduation	4	33.3	3	42.9	5	26.3	2	22.3
	**Total**	**12**	**100**	**7**	**100**	**19**	**100**	**9**	**100**
**4.**	**Occupation**								
	Employed	5	41.7	3	42.9	6	31.6	2	22.3
	Student	4	33.3	3	42.9	7	36.8	4	44.4
	House hold	3	25	1	14.2	6	31.6	3	33.3
	**Total**	**12**	**100**	**7**	**100**	**19**	**100**	**9**	**100**
**5.**	**Ethnicity**								
	Urdu speakers	4	33.3	4	57.1	7	36.8	3	33.3
	Sindhi	1	8.3	0	0	2	10.5	1	11.1
	Punjabi	3	25	1	14.3	4	21.1	1	11.1
	Pushtun	1	8.3	1	14.3	2	10.5	1	11.1
	Baloch	1	8.3	0	0	1	5.3	1	11.1
	Others	2	16.8	1	14.3	3	15.8	2	22.3
	**Total**	**12**	**100**	**7**	**100**	**19**	**100**	**9**	**100**

**Table 4:** Exploratory factor analysis (EFA) of BCI.

Serial Number	Research Questions	Factor						
		1	2	3	4	5	6	7
**1.**	Number of children	−0.954						
**2.**	Marital status	0.949						
**3.**	Occupation	0.535						
**4.**	Age	0.410	−0.212		−0.347			
**5.**	What would you advice to other women regarding the regarding BC screening?	0.214						
**6.**	Are you aware of physical self examination for breast cancer?		0.734					
**7.**	Did you ever perform physical self examination at home?		0.719					
**8.**	Did you ever discuss about breast cancer with your doctor?		0.670		0.202			
**9.**	Did you ever undergo breast screening at hospital or clinic?		0.462					
**10.**	Who did you encounter with breast cancer other than a family member?			0.901				
**11.**	Did you ever come across anyone other than family members who had breast cancer?			0.821				
**12.**	Most common symptom of breast cancer							
**13.**	Level of education				0.665			
**14.**	Family income				0.583			
**15.**	How do you feel about breast screening at hospital or clinic?				−0.405	0.238		
**16.**	Source of information about breast cancer and detection techniques				0.394			
**17.**	Ethnicity				0.391			
**18.**	Do you think BC is becoming a major problem of Pakistan?					0.680	−0.306	
**19.**	Are you aware of treatment for BC?					0.495	0.221	
**20.**	Are you aware of screening program for breast cancer?					0.490		
**21.**	Does being overweight/obesity inc risk?					0.417		
**22.**	Can BC spread to other parts of body?					0.321		
**23.**	How bad you would rate BC problem in Pakistan on a scale of 1–10?					−0.227	0.224	0.216
**24.**	Gender preference of health care provider when seeking breast cancer information or screening						−0.472	
**25.**	Are you aware of breast cancer?						0.418	
**26.**	Do you feel embarrassed talking about breast cancer in the society?						−0.366	
**27.**	Have you heard about self examination for BC?		0.235			0.274	0.314	
**28.**	What is the major risk factor for BC?							
**29.**	What is the reason for undergoing breast cancer screening in your opinion?							0.537
**30.**	If you ever undergo breast screening what would be your trigger factor?						−0.273	0.375
**31.**	Any family member having breast cancer?				0.274			−0.356
**32.**	What do you perceive as barriers to undergo breast screening?							

## Supplementary Materials

The following are available online at www.mdpi.com/2079-9721/4/4/37/s1.

## References

[1] Naz N., Khanum S., Dal Marcon Sasso G.T., de Lourdes Souza M.D. (2016). Women’s Views on Handling and managing their breast cancer in Pakistan: A qualitative study. Diseases.

[2] National Breast Cancer Foundation, USA. http://www.nationalbreastcancer.org/what-is-breast-cancer.

[3] Sohail S., Alam S.N. (2007). Breast cancer in Pakistan—Awareness and early detection. J. Coll. Physicians Surg. Pak..

[4] Badar F., Faruqui Z., Uddin N., Trevan E. (2011). Management of breast lesions by breast physicians in a heavily populated South Asian developing country. Asian Pac. J. Cancer Prev..

[5] Bhurgri Y. (2004). Karachi cancer registry data—Implications for the National Cancer Control Program of Pakistan. Asian Pac. J. Cancer Prev..

[6] News.com.au ‘Breast’ Ban Hinders Cancer Fight in Pakistan, 21 January 2014. http://www.news.com.au/lifestyle/health/challenge-rises-to-pakistans-breast-cancer-taboos/story-fneuz9ev-1226806297369.

[7] Gilani S.I., Khurram M., Mazhar T., Mir S.T., Ali S., Tariq S., Malik A.Z. (2010). Knowledge, attitude and practice of a Pakistani female cohort towards breast cancer. J. Pak. Med. Assoc..

[8] Sarwar M.Z.S., Shah H.S.F., Yousaf M.R., Ahmad Q.A., Khan S.A. (2015). Knowledge, attitude and practices amongst the Pakistani females towards breast cancer screening programme. J. Pak. Med. Assoc..

[9] Maqsood B., Zeeshan M.M., Rehman F., Aslam F., Zafar A., Syed B., Qadeer K., Ajmal S., Imam S.Z. (2009). Breast cancer screening practices and awareness in women admitted to a tertiary care hospital of Lahore, Pakistan. J. Pak. Med. Assoc..

[10] Ali A.A., Mobeen S., Mukhtar A., Syed N.U.A., Amer A., Noureen A. (2015). Breast cancer-risk factors. J. Rawalpindi Med. Coll. Stud..

[11] Sobani Z.U.A., Saeed Z., Baloch H.N.U.A., Majeed A., Chaudry S., Sheikh A., Umar J., Waseem H., Mirza M., Qadir I. (2012). Knowledge attitude and practices among urban women of Karachi, Pakistan, regarding breast cancer. J. Pak. Med. Assoc..

[12] Shaikh B.T., Haran D., Hatcher J. (2008). Women’s social position and health-seeking behaviors: Is the health care system accessible and responsive in Pakistan?. Health Care Women Int..

[13] National Breast Cancer Foundation, Inc., USA (2016). Symptoms and Signs. http://www.nationalbreastcancer.org/breast-cancer-symptoms-and-signs.

[14] Canadian Cancer Society (2016). Signs and Symptoms of Breast Cancer. http://www.cancer.ca/en/cancer-information/cancer-type/breast/signs-and-symptoms/?region=bc.

