# Supplementary material for: Developing a Research Instrument to Document Awareness, Knowledge, and Attitudes Regarding Breast Cancer and Early Detection Techniques for Pakistani Women: The Breast Cancer Inventory (BCI)
Source: Diseases. 2016 Dec 9;4(4):37. doi: 10.3390/diseases4040037 (PMC5456323; doi:10.3390/diseases4040037)
Supplement: Supplementary file 1 [file diseases-04-00037-s001.pdf]

Supplementary Materials: Developing a Research Instrument to Document Awareness, Knowledge, and Attitudes Regarding Breast Cancer, and Early Detection Techniques for Pakistani Women: The Breast Cancer Inventory (BCI)

Atta Abbas Naqvi, Fatima Zehra, Rizwan Ahmad and Niyaz Ahmad

Table S1. Scoring criteria for BCI.

Serial Number	Research Variable	Categories Available	Grading
**1.**	Do you know what breast cancer is?	Yes<br>No<br>Yes, but I am not sure	2<br>0<br>1
**2.**	What is the most common symptom of BC? ***	Established symptom according to identified standards	3
		(2) or more answers, all of which are established symptoms according to identified standards	3
		(2) answers, one of which is an established symptom according to identified standards and the other is not established, but has a likelihood	2
		(2) answers, one of which is an established symptom and the other is a wrong answer	1.5
		(1) or more answers, both of which are not established symptoms but are not wrong	1.5
		Answers which are neither established according to identified standards nor possess a likelihood i.e., wrong answers	0
**3.**	Do you know about the breast cancer screening?	Yes<br>No<br>Yes, but I am not sure	2<br>0<br>1
**4.**	Do you know about BC physical self examination?	Yes<br>No<br>Yes, but I am not sure	2<br>0<br>1

Table S1. *Cont.*

Serial Number	Research Variable	Categories Available	Grading
**5.**	Are you aware of mammography?	Yes	2
		No	0
		Yes, but I am not sure	1
**6.**	What is the most common risk factor of BC? ***	Established risk factors according to identified standards	3
		(2) or more answers, all of which are established risk factors according to identified standards	3
		(2) answers, one of which is an established risk factors according to identified standards and the other is not established but has a likelihood	2
		(2) answers, one of which is an established risk factor and the other is a wrong answer	1.5
		(1) or more answers, both of which are not established risk factors but are not wrong	1.5
		Answers which are neither established according to identified standards nor possess a likelihood i.e., wrong answers	0
**7.**	Are you aware of breast cancer treatment?	Yes	2
		No	0
		Yes, but I am not sure	1
**8.**	Can breast cancer spread to any other part of the body?	Yes	2
		No	0
		Yes, but I am not sure	1
**9.**	Can obesity increase the risk of breast cancer?	Yes	2
		No	0
		Yes, but I am not sure	1
**10.**	Do you know how to perform a BC physical self examination?	Yes	2
		No	0
		Yes, but I am not sure	1

*** Standards identified; National Breast Cancer Foundation USA [1], Canadian Cancer Society [2]).

References

1. National Breast Cancer Foundation, Inc., USA. Symptoms and signs. 2016. Available online: http://www.nationalbreastcancer.org/breast-cancer-symptoms-and-signs (accessed on 7 December 2016).
2. Canadian Cancer Society. Signs and symptoms of breast cancer. 2016. Available online: http://www.cancer.ca/en/cancer-information/cancer-type/breast/signs-and-symptoms/?region=bc (accessed on 7 December 2016).
